# Stable isotope analysis of ectoparasites as a tool for understanding trophic interactions with mammalian hosts

**DOI:** 10.1111/mve.70008

**Published:** 2025-08-27

**Authors:** Gaia G. Mortier, Stuart Black, Andrew C. Kitchener, Georg Hantke, Luke A. Stevens, Lea J. Grayston‐Smith, Phillip J. Baker, M. Alejandra Perotti

**Affiliations:** ^1^ Department of Geography and Environmental Science University of Reading Reading UK; ^2^ National Museums Scotland Edinburgh UK; ^3^ School of Geosciences Edinburgh UK; ^4^ School of Biological Sciences University of Reading Reading UK

**Keywords:** *Erinaceus europaeus*, European hedgehog, fat dormouse, fleas, *Glis glis*, Great Britain, *Ixodes hexagonus*, lice, red squirrel, *Sciurus vulgaris*, ticks

## Abstract

Climate change is expected to expand the geographic ranges of ectoparasites, increasing the transmission of vector‐borne diseases and necessitating a better understanding of ectoparasite–host trophic dynamics. Haematophagous ectoparasites can serve as valuable subsamples of their hosts, retaining isotopic values that reflect dietary information in both their blood meals and tissues. However, differences in the life histories and feeding strategies of lice, fleas and ticks may influence how host isotopic composition is preserved. Here, stable isotope values of carbon (*δ* ^13^C) and nitrogen (*δ* ^15^N) were used to investigate trophic interactions between ectoparasites and their mammalian hosts in three pairings: lice (Anoplura: Polyplacidae; *n* = 101) from Eurasian red squirrels *Sciurus vulgaris* L. (Rodentia: Sciuridae), fleas (Siphonaptera: Ceratophyllidae; *n* = 92) from fat dormice *Glis glis* L. (Rodentia: Gliridae) and ticks (Ixodida: Ixodidae; *n* = 16) from European hedgehogs *Erinaceus europaeus* L. (Eulipotyphla: Erinaceidae). Our findings indicate that ectoparasites reflect the dietary patterns of their hosts, with lice exhibiting the closest isotopic values, followed by fleas and ticks. All parasites had significantly higher *δ* ^15^N values than their hosts, indicative of trophic enrichment, but their *δ* ^13^C values varied. Notably, we found that the presence of a blood meal did not significantly affect the isotopic values found in lice and fleas, while ticks showed a significant difference between exoskeleton and blood meal in *δ* ^13^C values. This study highlights the importance of understanding how the life histories of parasite species influence the preservation of isotopic host signals in order to be able to utilise stable isotope analyses of ectoparasites to infer host dietary niches and preferences, with broader implications for understanding host–parasite dynamics and disease transmission pathways.

## Graphical abstract

- Ectoparasites showed significant δ^15^N enrichment compared to their hosts, indicating trophic enrichment, but δ^13^C preservation varied.
- Lice, fleas and ticks preserve their hosts' isotopic fingerprints to varying degrees, with lice reflecting host dietary patterns most closely, likely due to their exclusive, host‐specific feeding behaviour.
- The presence of blood meals did not significantly affect isotopic values in lice and fleas, but did in ticks, highlighting differences in isotopic signal preservation among parasite taxa.

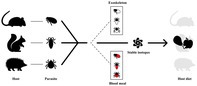

## INTRODUCTION

Despite their prevalence and role in the transmission cycle of vector‐borne diseases, the exact role of parasites within ecological food webs remains poorly understood (Sabadel et al., 2019; Thieltges et al., 2019). Identifying residual host biomarkers through external parasite (ectoparasite) tissue has greatly improved our understanding of complex ecosystem interactions and disease ecology, which is of growing importance due to projected climate change‐induced shifts in ectoparasite distributions (Cumming & Van Vuuren, 2006; Hamer et al., 2015). Stable isotope analysis has been proposed as one such method to address several limitations of molecular blood meal analysis as it offers information on previous hosts well beyond the limited window observed in DNA preservation and provides an opportunity to sample either blood meal or parasite exoskeleton tissue (Davey et al., 2007; Franklin et al., 2010; Graham et al., 2012; Hamer et al., 2015; Hendrick et al., 2019; Kent, 2009; Rasgon, 2008). Ectoparasites are valuable samplers of host tissues, and stable isotope ratios of ectoparasite tissues reflect those of their host (Butterworth et al., 2004; Jenkins et al., 2018; Melzer et al., 2020; Navock et al., 2019; Rasgon, 2008; Wang et al., 2024). However, despite their potential, isotopic studies on obligate ectoparasites that are commonly associated with humans and their pets, such as lice (Eder et al., 2023), fleas (Gómez‐Díaz & González‐Solís, 2010; Navock et al., 2019; Stapp & Salkeld, 2009) and ticks (Heylen et al., 2019; LoGiudice et al., 2018; Schmidt et al., 2011), remain underrepresented.

Stable isotope analysis uses naturally occurring stable isotopes, particularly those of nitrogen (^15^N/^14^N or *δ* ^15^N) and carbon (^13^C/^12^C or *δ* ^13^C), to examine dietary traces and the flow of nutrients between organisms (Rundel et al., 1989), with *δ* ^15^N and *δ* ^13^C values reflecting trophic levels and dietary sources of carbon, respectively (Newton, 2016; Post, 2002; Tieszen et al., 1983). As stable isotopes move through a food web, their ratios change and are incorporated into organic tissues through biochemical processes such as digestion, enabling researchers to use them to study food webs and trophic interactions (Rundel et al., 1989; Thieltges et al., 2019). Different rates of growth (or ‘turnover’ rates) observed in organic tissues cause them to incorporate isotopes over varying timespans. The difference (*Δ*) between stable isotope ratios of nitrogen (*Δ* ^15^N) and carbon (*Δ* ^13^C) between consumers (e.g., parasites) and their diet (e.g., host tissues) is the trophic discrimination factor (TDF) (Newton, 2016).

**FIGURE 1:**
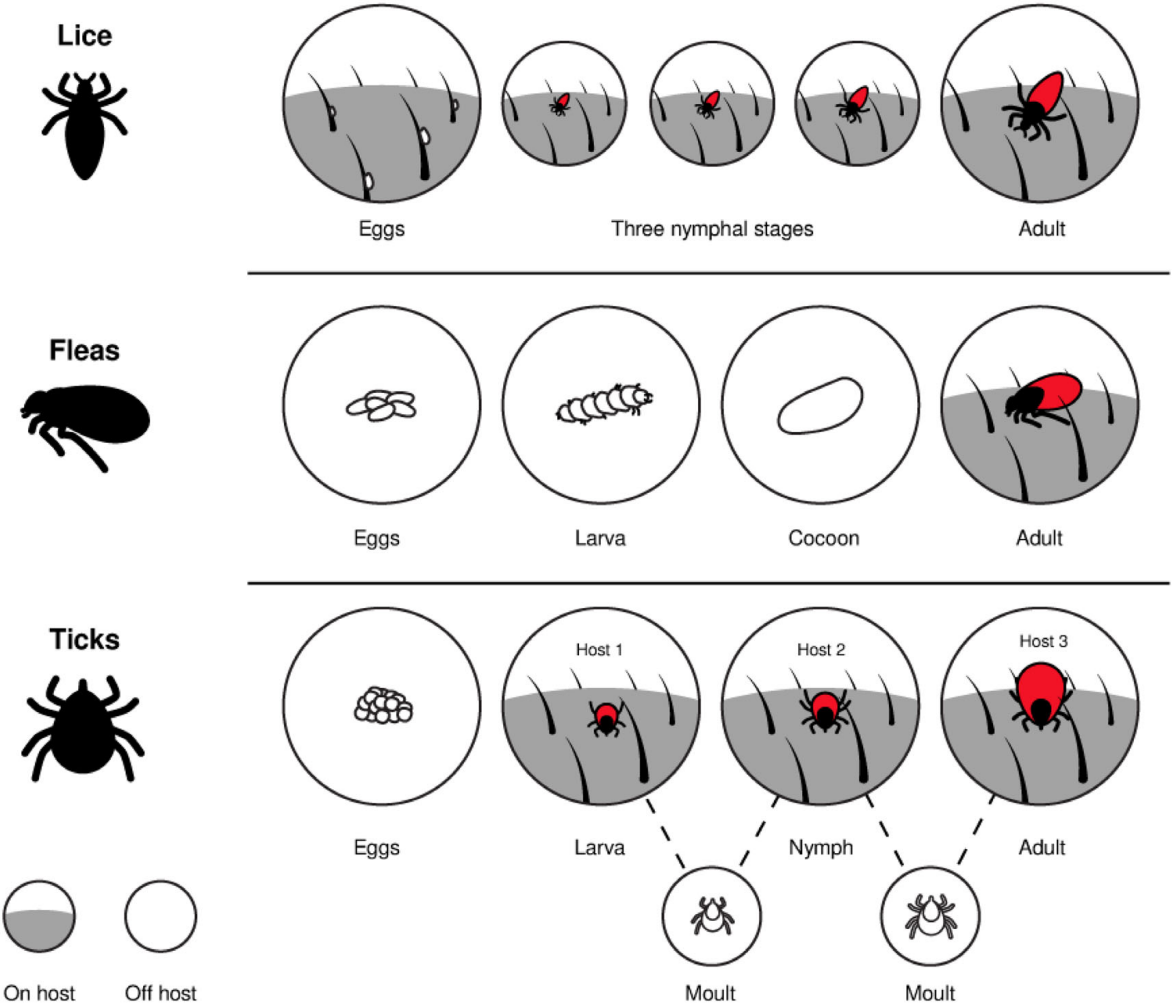
Generalised diagram of the life stages of lice, fleas and ixodid ticks, showing the periods during which the parasites are present on the host. Lice spend their entire life cycles on hosts as the larvae of lice hatch from eggs attached to host fur or feathers before undergoing several nymphal stages, followed by a final adult stage. By contrast, fleas spend most of their life cycle in their host's environment as opposed to on their host directly. Flea eggs are laid onto a host to drop off into the immediate surroundings (usually a host's nest or den), where they develop until free‐living larvae hatch that mainly feed on detritus. The flea larvae then pupate inside cocoons prior to entering their adult stage, during which they seek out hosts to obtain a blood meal and mate. Ixodid ticks undergo a three‐host life cycle, where eggs are laid into the environment from which larvae hatch. Before the larvae moult into nymphs, they obtain their first blood meal, before dropping off and finding their next host. Nymphs feed on another blood meal in order to reach the adult stage, during which the ticks feed one final time before mating and dropping off (based on Apanaskevich & Oliver Jr., 2014; Lehane, 1991).

There are several key differences in the lifestyles of lice, fleas and ticks, which are important to consider when investigating trophic interactions with their mammalian hosts (Figure 1). Parasitic lice (Psocodea: Phthiraptera) are permanent obligate parasites and spend their entire life cycle on a single host, only moving to different individuals of the same species under specific conditions (Ferris, 1951; Lehane, 1991; McGavin, 2001; Rothschild & Clay, 1952). Accordingly, lice are highly host‐specific and have been co‐evolving with, and becoming specialised on, their hosts (Marshall, 1981; McGavin, 2001). Fleas (Insecta: Siphonaptera) are intermittent feeders that prefer the blood of mammals but are also known to feed on birds (Rothschild & Clay, 1952; Whitaker, 2007). Although most flea species have distinct host preferences (i.e., their ‘true host’), they will feed on other animals when their preferred host is unavailable, although this can lead to a loss in fecundity (Lehane, 1969; Marshall, 1981; McGavin, 2001). Generally, fleas are associated with animals living in nests or dens, as their free‐living larvae feed off the organic detritus from the host (Rothschild & Clay, 1952; Whitaker, 2007). Hard (or ixodid) ticks (Acari: Ixodidae) feed on the blood of mammals, birds, reptiles and amphibians (Russell et al., 2013). Generally, ixodid ticks undergo a three‐host life cycle, relying on different host species during each life stage (Sonenshine, 1991). Ticks can be nidicolous, seeking hosts near nests, or non‐nidicolous, seeking hosts in open spaces like grasslands; nidicolous ticks are more likely to specialise on hosts within the same area (Apanaskevich & Oliver Jr., 2014; Pfäffle et al., 2011).

Sucking lice feed on blood throughout all their life stages. Most lice remain permanently on their host, feeding multiple times a day. Over the course of 5–30 min, lice take up small blood meals (20%–30% of unfed body weight), which they digest almost immediately after feeding, repeating the process every 2 h (Lambiase & Perotti, 2019; Lehane, 1991; Marshall, 1981). As a result, the blood meal in lice represents the last ~30 min of feeding and is likely to reflect the stable isotope values of a singular host. Unlike lice, fleas only feed on blood once they have reached adulthood (McGavin, 2001). Fleas feed once or twice a day, although the duration and frequency of feeding depend greatly on species and mating status (Lehane, 1969; Marshall, 1981). Generally, the more frequently a flea feeds, the smaller the blood meal, whereas fleas that feed on their host for extended periods of time (up to 4 h) take larger blood meals (Marshall, 1981). Blood meals in fleas are usually retained for up to 24 h, during which they may switch hosts, meaning that their stable isotope values are more likely to reflect multiple individuals (Lehane, 1991). By contrast, ticks are likely to isotopically reflect a single host for a longer period, as they feed infrequently but for extended periods of time and take up substantial blood meals relative to their body size (Russell et al., 2013). A single feeding session can last as long as 15 days in mated adult females and up to 12 days in unmated females (Toutoungi et al., 1993). After feeding, ticks drop off the host to digest and moult or, in the case of mated female adults, lay eggs after having a final feeding stage called the ‘big sip’ (Russell et al., 2013; Sonenshine, 1991; Toutoungi et al., 1995).

This study investigated the trophic interactions between ectoparasites (lice, fleas and ticks) and their mammalian hosts within a stable isotope ecology framework. Mammals host a range of arthropod ectoparasite species, including numerous species of fleas, lice and ticks (Lehane, 1991; Marshall, 1981). We focused on three mammalian hosts, selected to represent a range of stable, distinct diets and their associated ectoparasites: (i) the Eurasian red squirrel *Sciurus vulgaris* L. (Rodentia: Sciuridae); (ii) fat or edible dormouse *Glis glis* L. (Rodentia: Gliridae) and (iii) European hedgehog *Erinaceus europaeus* L. (Eulipotyphla: Erinaceidae). The fat dormouse was imported to Britain from mainland Europe in 1902 and is primarily found in Hertfordshire, close to its original introduction site (Morris and Morris, 2010). Its diet varies seasonally, primarily consisting of seeds, fruit, plant buds, occasionally bird eggs and nestlings and importantly European beech tree *Fagus sylvatica* L. (Fagales: Fagaceae) masts, upon which it is dependent for sexual reproduction (Juškaitis et al., 2015; Morris and Morris, 2010; Parrott et al., 2008). Within Great Britain, red squirrels are largely confined to parts of Scotland, Northern England, Northern Ireland and isolated areas in Wales and Southern England, following population declines driven by the introduction of grey squirrels *Sciurus carolinensis* Gmelin, 1788 (Rodentia: Sciuridae) (Gurnell, 1987; Mathews et al., 2018). Their diet primarily includes seeds, but they are also known to eat berries, mushrooms and occasionally bird eggs (Krauze‐Gryz & Gryz, 2015). European hedgehogs are found throughout Great Britain, where they are most common in urban and suburban areas, though their abundance and range have declined significantly since the late 20th century (Burton, 1969; Hof, 2009; Mathews et al., 2018; Schaus et al., 2020; Wembridge et al., 2022). Their seasonally varied diet primarily includes invertebrates such as insects, worms and, to a lesser extent, slugs and snails, with urban hedgehogs potentially consuming supplementary food provided by humans (Reeve, 1994; Schaus et al., 2020; Wroot, 1984). The aims of this study were to estimate trophic discrimination factors (TDFs) for each ectoparasite–host pair and assess whether ectoparasite isotope values reflect those of their mammalian hosts. Our a priori hypothesis was that the stable isotope values of lice would most closely resemble those of their host, given the life histories of each study species. Our findings will improve the application of ectoparasites as proxies for analysing host biochemical profiles in both ecological and epidemiological research.

## METHODS

### Study species and specimen collection

Ectoparasites were removed from (i) fat dormice that were collected from Hockeridge Wood (51°45′N, 0°40′W), Hertfordshire, England, in July 2019; (ii) red squirrels from Formby, Lancashire, in 2019 and (iii) live European hedgehogs (*n* = 5) that were accessed via Herbies Hedgehog Rescue, Berkshire, England, in April 2023 (Table S1). Museum skins of fat dormice and red squirrels are stored in the collections of National Museums Scotland. For a subset of the museum specimens, muscle tissue, fur and whiskers were sampled from Eurasian red squirrel (*n* = 2) and fat dormouse (*n* = 1) specimens. Ectoparasites and host tissues were then kept frozen until further analysis. No permits or ethical approvals were required for this study as no fieldwork or handling of live animals was conducted. Red squirrel and fat dormice samples were obtained from archival specimens held by National Museums Scotland, with sampling carried out under formal loan agreements (NS.OL.2023.55 and NS.OL.2023.22). Hedgehog spines and associated ectoparasites were collected by the wildlife centre as part of routine animal care. All applicable international, national and institutional guidelines for the care and use of animals were followed.

#### Ectoparasite identification

Lice (*n* = 154) collected from Eurasian red squirrels (*n* = 2) were identified using Ferris (1951), with primary characteristics used for identification being the (thoracic) sternal plate and head shape. Fleas (*n* = 88) obtained from fat dormice (*n* = 25) were identified using Whitaker (2007). Ticks from hedgehog hosts (*n* = 10) were identified using Arthur (1963), with identification characteristics including the size and prominence of the scutum. A subset of louse (*n* = 54) and flea (*n* = 76) individuals were categorised as ‘fed’ or ‘unfed’ (i.e. the presence of absence of a blood meal), following Stapp and Salkeld (2009). An additional set of female ticks (*n* = 6) belonging to one hedgehog host was dissected to separately analyse the blood meal and exoskeleton tissues. Blood meals were separated from exoskeletons using stainless steel pointed forceps and a scalpel.

### Stable isotope analysis

Individual ectoparasites (fleas, lice, ticks) were rinsed three times using ultra‐high‐quality water (17 MΩ UHQ; hereafter UHQ water), produced in‐house. Tick exoskeletons were placed within a 1.5‐mL tube containing UHQ water before being centrifuged at 8500*g* for 1 min, followed by vortexing for 5 s prior to the removal of the waste solution, to ensure that all remaining blood meal was removed. This process was repeated until the waste solution was clear (2–3 repeats). Whole ectoparasites, as well as tick exoskeletons and blood meals, were placed in separate 1.5‐mL tubes and frozen prior to undergoing a 48‐h freeze‐drying process.

Lipids and external contaminants were removed from host muscle tissue, fur, whiskers and spines using chloroform: methanol solution (2:1 v:v) and repeated UHQ water rinses. Samples were then dried at 40°C for a minimum of 48 h and frozen for ≥48 h before undergoing a 48‐h freeze‐drying process. Freeze‐dried muscle tissue, whole ticks and tick blood meals were homogenised using a mortar and pestle until they resembled a fine powder. Fur, whisker and tick exoskeleton samples were subsampled using tweezers and a scalpel, whereas hedgehog spines were chronologically sub‐sectioned to 1‐mm slices using tweezers and micro‐scissors. All samples were weighed in triplicate (aliquots ranging from 0.15 to 0.25 mg) into tin capsules (6 × 4 mm), using a Sartorius™ Cubis MSA6.6S‐000‐DM microbalance (Sartorius AG, Göttingen, Germany). For lice and fleas, some individuals corresponding to the same host were pooled (2–6 individuals) to assure that the samples met the minimum mass for reliable isotope analysis. The stable isotope ratios of carbon and nitrogen were measured using a DeltaV Advantage isotope ratio mass spectrometer coupled with an Isolink CNSOH Elemental Analyzer (Thermo Fisher Scientific, Bremen, Germany) at the Chemical Analysis Facility at the University of Reading. The findings are presented as *δ* values per mil (‰) relative to the international standards Vienna PeeDee Belemnite (VPDB) for carbon and atmospheric N_2_ (AIR) for nitrogen. Data were adjusted for linearity and instrument by analysing an in‐house standard every five samples and stretch corrected by analysing in‐house standards (x4) and international standards (x4, USGS‐56, USGS‐61, USGS‐62 and USGS‐63). Comparisons between measured values of internal and international standards against expected values were conducted to determine analytical error, which was found to be <0.15 ‰ for *δ* ^13^C and *δ* ^15^N.

### Data analysis

All statistical analyses were performed using Minitab 21.0.0. Parasite *δ* ^15^N and *δ* ^13^C values were assessed for the assumptions of normality and homogeneity of variances using Shapiro–Wilk's or Anderson‐Darling and Levene's tests, respectively. First, for the subset of samples for which information was available, a linear mixed‐effects model (LMM) was used to assess whether parasite *δ* ^15^N and *δ* ^13^C values varied according to the presence of a blood meal. Parasite species and the two‐way interaction also were included in this LMM. Host ID was included as a random effect to account for multiple parasites being sampled from the same host. As the assumptions of normality were violated (Shapiro–Wilk's tests, *p* < 0.05), the *δ* ^15^N and *δ* ^13^C values were transformed (log_10_ + 30 and log_10_, respectively) prior to analysis. Comparisons between parasite *δ* ^15^N or *δ* ^13^C values collected from a single host were made using a two‐sample *t* test, after the assumptions were satisfied (Anderson‐Darling tests, *p* < 0.05).

TDFs (*Δ* ^15^N or *Δ* ^13^C) were calculated for each ectoparasite–host individual pairing (each tissue analysed separately) according to the equation: *Δ* ^15^N or *Δ* ^13^C = *δ*X_parasite_ – *δ*X_host_, where *δ*X_parasite_ and *δ*X_host_ represent the *δ* ^15^N or *δ* ^13^C values of the parasite and host tissue (fur, whisker and muscle), respectively. TDFs were calculated separately for ‘fed’ and ‘unfed’ lice and fleas and for the blood meal and exoskeleton of ticks. Finally, LMMs were used to investigate whether parasite *δ* ^15^N and *δ* ^13^C were significantly related to those of host tissues. Host ID was included as a random effect. In addition, a linear regression analysis was performed to model the relationship between parasite *δ* ^15^N and *δ* ^13^C values in relation to host tissue types and parasites as predictors, with nitrogen and carbon as response variables. In some instances, there were not enough data points available for a pairwise approach, and instead mean values were used. Significance was assumed at α = 0.05 in all cases, and unless otherwise stated, values are reported as mean ± standard deviation.

## RESULTS

### Ectoparasite species identified

The prevalent louse species collected from Eurasian red squirrels was *Neohaematopinus sciuri* (Packard, 1891) (Anoplura: Polyplacidae). The vast majority (95%) of fleas from fat dormice were the grey squirrel flea *Orchopeas howardi howardi* (Baker, 1895) (Siphonaptera: Ceratophyllidae), with only a small number of other species being represented by one or two individuals: *Ctenophthalmus nobilis nobilis* (Rothschild, 1900), *C. nobilis vulgaris* (Jordan & Rothschild, 1912), *Ceratophyllus hirundinis* (Curtis, 1826) and *Doratopsylla dasycnema dasycnema* (Rothschild, 1902) (all Siphonaptera: Ceratophyllidae). All ticks collected from hedgehogs were engorged adult females of the species *Ixodes hexagonu*s Leach, 1815 (Ixodida: Ixodidae), also known as the hedgehog tick.

### Effect of blood meal on parasite values

**FIGURE 2:**
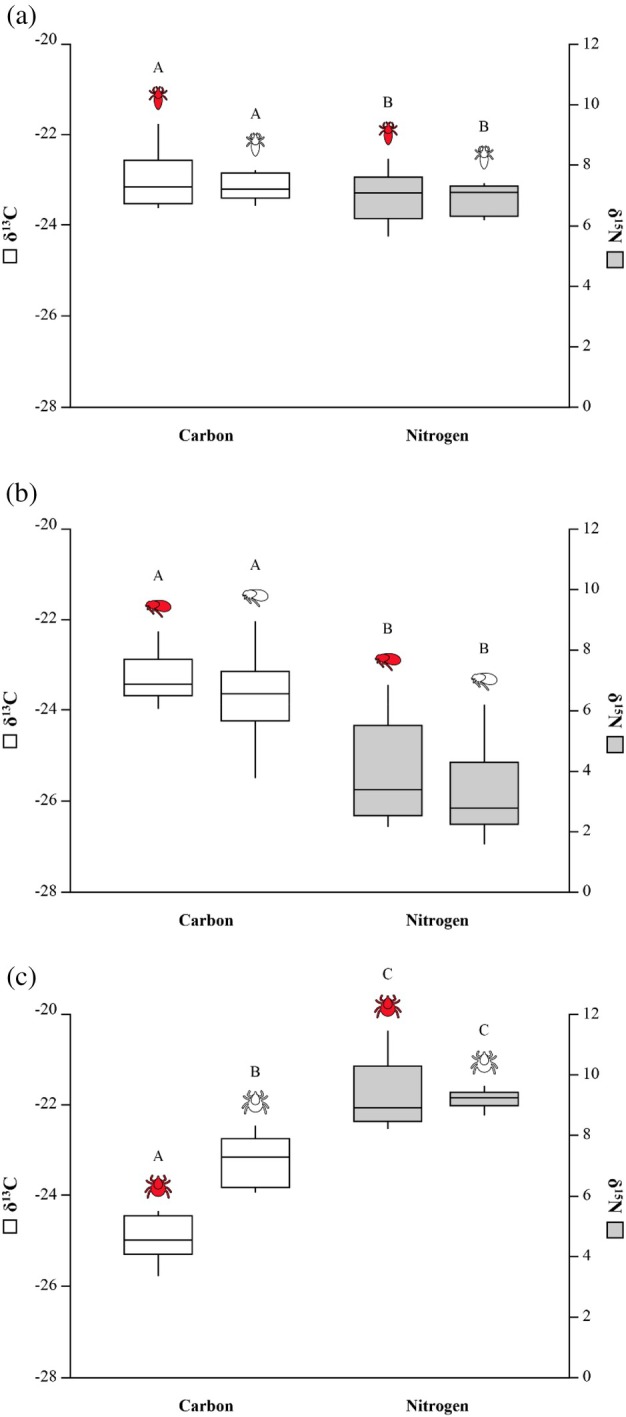
Nitrogen and carbon isotope ratios (‰) in parasite blood meals and exoskeletons. Mean *δ* ^13^C (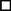) and *δ* ^15^N (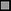) in ‰ between fed (in red) and unfed (in white) (a) lice (*n* = 55; 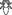), (b) fleas (*n* = 169; 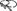) and (c) ticks (*n* = 10; 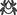), with Tukey grouping showing significance.

No significant difference was observed between fed (*δ* ^15^N: 6.9 ‰ ± 0.8; *δ* ^13^C: −23 ‰ ± 0.6) and unfed (*δ* ^15^N: 6.8 ‰ ± 0.5; *δ* ^13^C: −23.2 ‰ ± 0.3) lice for both *δ* ^15^N (two‐sample *t* test: *t*_19_ = 0.35, *p* = 0.727) and *δ* ^13^C (*t*_18_ = 0.78, *p* = 0.447; Figure 2a; Table S2). In fleas, results of the mixed‐effects model analysis showed no significant difference between fed (*δ* ^15^N: 3.9 ‰ ± 1.7; *δ* ^13^C: −23.3 ‰ ± 0.5) and unfed (*δ* ^15^N: 3.3 ‰ ± 1.4; *δ* ^13^C: −23.7 ‰ ± 0.9) specimens for both *δ* ^15^N (LMM, estimate (β) ± SE = −0.03 ± 0.025, *p* = 0.247) and *δ* ^13^C (−0.021 ± 0.011, *p* = 0.075; Figure 2b). By contrast, a significant difference was found between tick blood meals (*δ* ^15^N: 9.5 ‰ ± 1.2; *δ* ^13^C: −25.9 ‰ ± 0.2) and exoskeletons (*δ* ^15^N: 9.3 ‰ ± 0.3; *δ* ^13^C: −24 ‰ ± 0.2) for *δ* ^13^C (two‐sample *t* test: *t*_20_ = −8.95, *p* < 0.001) but not for *δ* ^15^N (*t*_20_ = 0.66, *p* = 0.519). Tick blood meals showed a wider range of *δ* ^15^N values than exoskeletons, with the latter showing less negative *δ* ^13^C values (Figure 2c).

### Variation in parasite–host isotope values

**FIGURE 3:**
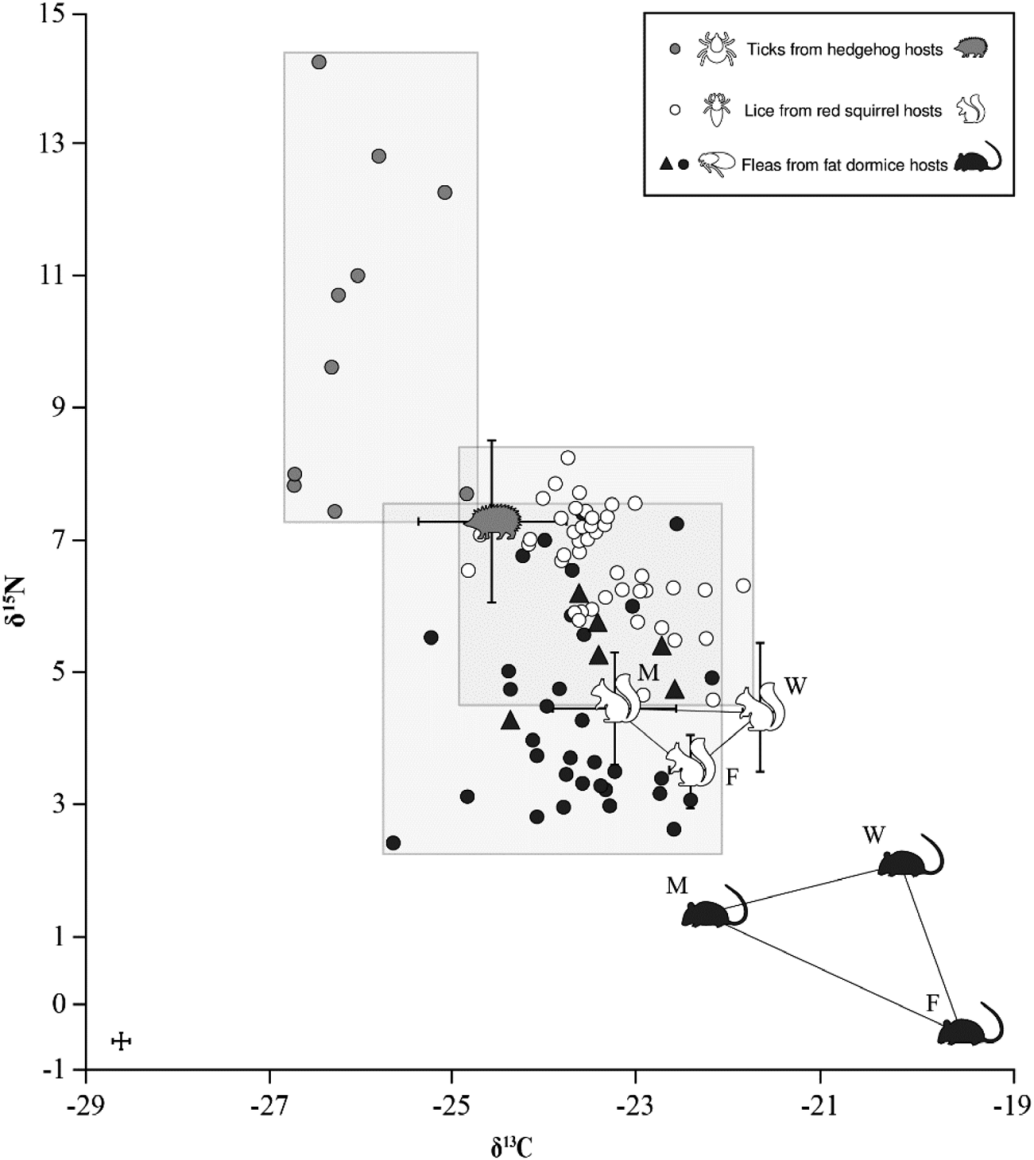
Nitrogen and carbon isotope ratios (‰) of lice, fleas and ticks, with tissues of their hosts: red squirrels, fat dormice and hedgehogs. Mean *δ* ^15^N and *δ* ^13^C (in ‰) of lice (*n* = 156) from red squirrels [muscle (M), whisker (W), fur (F), ±SD, *n* = 2], fleas (*n* = 66) from various fat dormice hosts, fat dormouse muscle (M), whisker (W), fur (F) (*n* = 1; fleas from this individual fat dormouse in triangles; *n* = 16) and ticks (*n* = 10) from hedgehogs (spines, ±SD, *n* = 5).

As blood meal presence was found to have no impact on *δ* ^15^N or *δ* ^13^C values in fleas or lice, these groups were combined for subsequent calculations. However, ticks were analysed separately. Host tissue values and offsets are provided in the supplementary information (Table S3). The average parasite–host difference between lice and their host tissues was *Δ* ^15^N_Host‐Louse_ 1.8 ‰ ± 0.6 and *Δ* ^13^C_Host‐Louse_ −0.3 ‰ ± 0.8 (Figure 3; Table S4). According to the LMM, the *δ* ^15^N values of lice were significantly higher than those of fur (*Δ* ^15^N_Fur‐Louse_ 2.4 ‰ ± 0.4, −1.087 ± 0.141, *p* < 0.001) and whiskers (*Δ* ^15^N_Whisker‐Louse_ 1.4 ‰ ± 0.4, 1.431 ± 0.127, *p* < 0.001), but not those of muscle (*Δ* ^15^N_Muscle‐Louse_ 1.4 ‰ ± 0.2, −0.109 ± 0.155, *p* = 0.486). Lice were most similar in *δ* ^13^C values to those of their host's fur (*Δ* ^13^C_Fur‐Louse_ −0.4 ‰ ± 0.2, 0.117 ± 0.103, *p* = 0.259) but were significantly less positive than those of host whiskers (*Δ* ^13^C_Whisker‐Louse_ −1.1 ‰ ± 0.1, −0.239 ± 0.092, *p* = 0.012) and muscle tissue (*Δ* ^13^C_Muscle‐Louse_ 0.5 ‰ ± 0.2, −0.709 ± 0.113, *p* < 0.001). Notably, the smallest analysed louse samples, which contained an individual louse and weighed 0.035 mg, still provided robust isotopic values.

A small number of outliers (*n* = 5) was observed among the fleas (*n* = 16) from one fat dormouse specimen, which showed average isotopic values of 3.3 ‰ ± 0.5 (*δ* ^15^N) and −20 ‰ ± 0.5 (*δ* ^13^C). These values differed significantly from those of the rest of the fleas for both *δ* ^15^N (4.8 ‰ ± 0.7; two‐sample *t* test: *t*_7_ = −3.27, *p* = 0.014) and *δ* ^13^C (−23 ‰ ± 0.65; *t*_7_ = 8.6, *p* < 0.001) and were dissimilar to the values of the fleas obtained from other fat dormouse hosts (*δ* ^15^N 3.8 ‰ ± 1.5; *δ* ^13^C −23.5 ‰ ± 0.74). In fleas, the average parasite–host values were *Δ* ^15^N_Host‐Flea_ 3.8 ‰ ± 1.4 and *Δ* ^13^C_Host‐Flea_ −2.6 ‰ ± 1.46, whereas the outlier fleas on average showed *Δ* ^15^N_Host‐Flea_ 2.3 ‰ ± 1.4 and *Δ* ^13^C_Host‐Flea_ −0.7 ‰ ± 1.46. These outliers were excluded from further analyses. Flea *δ* ^15^N values were most similar to those of their fat dormouse host's whiskers (*Δ* ^15^N_Whisker‐Flea_ 2.7 ‰; two‐sample *t* test: *t*_2_ = 3.22, *p* = 0.085) as they significantly differed from those of the muscle tissue (*Δ* ^15^N_Muscle‐Flea_ 3.5 ‰; *t*_13_ = 8.07, *p* = 0). For *δ* ^13^C values, fleas showed the closest similarity to their host's muscle tissue (*Δ* ^13^C_Muscle‐Flea_ −1 ‰; two‐sample *t* test: *t*_13_ = 0.54, *p* = 0.599) while being significantly different to those of the hosts' whiskers (*Δ* ^13^C_Whisker‐Flea_ −3.1 ‰; *t*_5_ = −2.75, *p* = 0.04). Flea *δ* ^15^N and *δ* ^13^C values were most dissimilar from those of host fur (*Δ* ^15^N_Fur‐Flea_ 5.3 ‰; *Δ* ^13^C_Fur‐Flea_ −3.8 ‰) compared to muscle tissue and whiskers. Here, it is of note that in the case of fat dormouse fur samples, three replicates were analysed, yet only one yielded reliable data for statistical analysis. Ticks exhibited greater variability in *δ* ^15^N values than lice and fleas. The LMM revealed that ticks had significantly higher nitrogen (*Δ* ^15^N_Spine‐Tick_ = 2.7 ‰ ± 1.61, −1.358 ± 0.348, *p* = 0.017) and more negative carbon (*Δ* ^13^C_Spine‐Tick_ = −1.3 ‰ ± 0.8, 0.647 ± 0.179, *p* = 0.022) isotope values than hedgehog host spines.

### Parasite–host linear relationships

**FIGURE 4:**
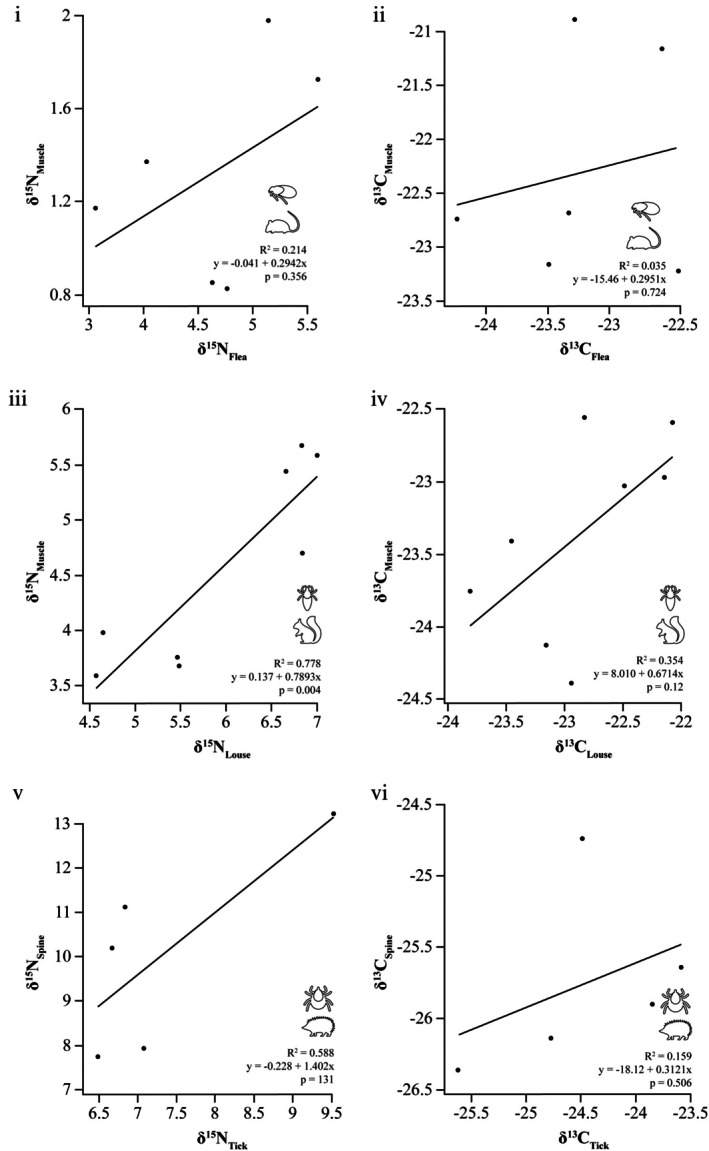
Regression lines of nitrogen and carbon isotope ratios (‰) between lice, fleas, ticks and their hosts: red squirrels, a fat dormouse and hedgehogs. Relationships between *δ* ^15^N (left) and *δ* ^13^C (right) in ‰ of (i, ii) fat dormouse (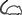) muscle tissue and fleas (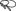), (iii, iv) red squirrel (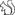) muscle tissue and lice (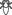) and (v, vi) hedgehog (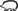) spines and ticks (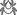), including regression lines.

Regression analyses showed a significant positive linear relationship between red squirrel muscle tissue and lice for *δ* ^15^N (*R* ^2^ = 0.778; *p* = 0.004) but not for *δ* ^13^C (*R* ^2^ = 0.354; *p* = 0.12). No significant linear relationship between fat dormouse muscle tissue and fleas for either *δ* ^15^N (*R* ^2^ = 0.214; *p* = 0.356) or *δ* ^13^C (*R* ^2^ = 0.035; *p* = 0.724) was evident (Figure 4). Similarly, no significant linear relationship was observed between hedgehog spines and ticks for *δ* ^15^N (*R* ^2^ = 0.588; *p* = 0.131) or *δ* ^13^C (*R* ^2^ = 0.159; *p* = 0.506).

## DISCUSSION

Our findings indicate that ectoparasites mirror their hosts' isotopic patterns, with lice, fleas and ticks each showing varying degrees of similarity to those of their mammalian hosts. We observed a consistent increase in *δ* ^15^N in ectoparasites compared to their hosts, as expected due to trophic enrichment (Jenkins et al., 2018; Post, 2002). This enrichment, combined with the observed variability in *δ* ^13^C, is in agreement with findings from previous studies (e.g., Butterworth et al., 2004; Melzer et al., 2020; Wang et al., 2024), though they do not align with values commonly associated with predator–prey relationships – around 1 ‰ for *δ* ^13^C and 3 ‰ for *δ* ^15^N (Post, 2002). Owing to their distinct life cycles and feeding strategies, lice, fleas and ticks preserve their hosts' isotopic fingerprints to varying degrees.

Lice are obligatory haematophagous and permanent ectoparasites and showed the greatest isotopic resemblance to their hosts in both *δ* ^15^N and *δ* ^13^C values compared to fleas and ticks. This agrees with Eder et al. (2023), who reported significant nitrogen enrichment, but no significant differences in *δ* ^13^C between lice and their hosts. Lice derive all their nutrients from a single, individual host, whereas fleas and ticks frequently feed on multiple hosts, resulting in greater variability in their isotopic values (Lehane, 1991; Marshall, 1981). Reflecting this difference in feeding behaviour, we found a significant positive linear relationship between red squirrel muscle tissue and lice for *δ* ^15^N, indicating that lice are reliable indicators of their host's nitrogen isotopic composition. However, no such relationship was observed for *δ* ^13^C, suggesting that lice do not consistently represent their hosts' carbon isotope values. Fleas and ticks showed no linear correlation with their hosts' muscle tissue and spines, respectively, for either *δ* ^15^N or *δ* ^13^C. Studies analysing parasite feeding relationships often do not take carbon isotope ratios into consideration, with Stapp and Salkeld (2009) reporting that while an overall increase in *δ* ^15^N was seen between fleas and their hosts, *δ* ^13^C values did not provide much information regarding the parasite–host feeding relationship (Rasgon, 2008; Thieltges et al., 2019). However, our results revealed a subgroup of flea outliers based on their *δ* ^13^C values, suggesting that these fleas potentially reflected host dietary specialisation or had fed on different hosts than those they were collected from, which has implications for interpreting isotopic parasite–host relationships in multi‐host systems. This potential host switching is facilitated by the sharing of habitats and occupation of grey squirrel nests by fat dormice, which could explain the high abundance of the grey squirrel flea (*O. howardi howardi*) observed in analysed fat dormice specimens (George, 2008; Smit, 1957; Whitaker, 2007). A similar phenomenon is known to occur in ticks, where hedgehog populations, now largely restricted to urban and suburban environments, form higher density populations than in rural areas (Hubert et al., 2011; Schaus et al., 2020). These urban settings may contribute to increased parasite prevalence and host transfer (Baptista et al., 2023; Gaglio et al., 2010).

Our findings demonstrate that the presence of a visible blood meal in lice and fleas did not significantly impact their isotopic values. Fed fleas were higher in *δ* ^15^N and *δ* ^13^C values than unfed fleas, while isotope values of fed lice were minimally different to those of unfed lice. As lice feed exclusively on one host, these minimal differences in their isotopic values are likely due to a constant diet. For fleas, these findings indicate a high level of host‐species specificity, as their recent blood meals align with the isotopic values of their exoskeletons, reflecting a preference for a host species rather than individual hosts. By contrast, ticks showed a significant difference between *δ* ^13^C values of exoskeleton and blood meal but not for *δ* ^15^N values. This significant difference in carbon isotope values between tick exoskeletons and blood meals could be indicative of different hosts or environments during previous life stages, but it may also result from tissue‐specific isotopic offsets or the influence of lipids in the blood meal which are known to show lower *δ* ^13^C values (Gómez‐Díaz & González‐Solís, 2010). Here, it is important to note that ticks have a significantly larger blood meal‐to‐body size ratio than lice and fleas, making their isotopic values more heavily influenced by the blood meal, whereas smaller ectoparasites are less impacted. All fed ectoparasites showed a wider range of isotope values than their unfed (exoskeleton) counterparts, which could be indicative of digestion occurring at different rates within the parasite blood meal (Hamer et al., 2015).

The interpretation of the isotopic offsets between parasites and hosts depends on several factors, including host tissue, as well as parasite life stage, sex, taxon and type of nutrients being metabolised (Eder et al., 2023; Neilson et al., 2005). While the analysed host tissues did not include the blood directly consumed by the ectoparasites, previous studies have shown that mammalian blood isotope values closely resemble those of muscle tissue (Barath et al., 2021; Roth & Hobson, 2000). Gómez‐Díaz and González‐Solís (2010) demonstrated that fleas and lice showed significantly increased *δ* ^15^N and *δ* ^13^C values compared to their hosts' blood, indicating that the isotopic values of blood would likely be more negative than those of the analysed muscle tissue. In both dormouse and red squirrel samples, muscle tissue was most similar to whiskers in *δ* ^15^N values, whereas whiskers were more similar to fur in *δ* ^13^C values. Hedgehog spines had significantly higher *δ* ^15^N values than dormice and red squirrel tissues, while their *δ* ^13^C values were overall lower. This is likely due to their insectivorous diet or the supplementary feeding provided by humans, both of which are likely to result in high *δ* ^15^N values (Herrera et al., 2001; Schaus et al., 2020).

Although the sample size for host tissues was too small to determine tissue discrimination factors, the consistent isotopic differences observed are likely due to tissue‐specific characteristics rather than dietary variations, given the stability of the hosts' diets (Juškaitis et al., 2015; Krauze‐Gryz & Gryz, 2015; Wroot, 1984). As fur grows approximately 1 cm per month, each centimetre represents an average of dietary isotopic values for approximately 4 weeks (Huelsemann et al., 2009). Whiskers have been observed to grow slightly faster (1.5 cm/month), whereas muscle tissue is known to reflect dietary intake over a period of 2–3 months (Cecchetti et al., 2021; Sponheimer et al., 2006). Rigid, slow‐growing tissues like hedgehog spines are believed to reflect extended durations, although exact timeframes are unknown (Tynes, 2010). Blood is constantly synthesised at a fast rate, allowing for a detailed dietary reflection within the last 24 h (plasma) or over a couple of weeks (red blood cells) (Newton, 2016). Therefore, blood meals preserved within ectoparasites are likely to reflect short‐term dietary patterns. Arthropod exoskeletons are grown only during a moult, a process that occurs between life stages and takes around 1–2 days in lice and fleas and 1–2 weeks in ticks (Lehane, 1991; Sonenshine, 1991). Once formed, exoskeletons remain inert and form an isotopic average of the prior feeding period.

Owing to their host‐specificity, lice are more likely to contain both an exoskeleton and a blood meal profile that isotopically reflects their most recent host. By contrast, fleas develop their adult exoskeletons during the larval stage, incorporating stable isotopes from the organic detritus in their host's nest or den (Stapp & Salkeld, 2009). Interestingly, this suggests that flea exoskeletons may accurately reflect the isotopes of their ‘true host’ as fleas do not often successfully reproduce on animals other than their preferred host (Lehane, 1991; Rothschild & Clay, 1952). Similarly, tick exoskeletons isotopically reflect their feeding as a previous life stage, with larvae preserving the stable isotope ratios from the maternal blood meal taken by the female prior to laying eggs (Sipari et al., 2024).

Overall, our results show that different species of ectoparasites represent their hosts' isotopic signature in distinct ways. We found that *δ* ^15^N isotope values were significantly higher in ectoparasites than in their hosts, while the *δ* ^13^C values exhibited greater variability. Lice most closely resembled their hosts' isotopic values likely due to their host‐specific and obligate parasitic lifestyle. By contrast, fleas and ticks showed more variable isotopic values and no significant linear correlation with their hosts' isotopic patterns, indicating a higher likelihood of feeding on multiple hosts. These interspecies variations suggest that host specificity and the isotopic turnover of ectoparasite tissues play an important part in the retention of host isotopic values. Isotopic approaches to parasitology could have potential applications in understanding parasite–host dynamics and improving vector‐borne disease transmission models. Future research should include geographical variability and multi‐host interactions, alongside controlled feeding studies, in order to establish ectoparasites as reliable indicators of their hosts' isotopic values.

## AUTHOR CONTRIBUTIONS

**Gaia G. Mortier:** Conceptualization; methodology; writing – original draft; investigation; formal analysis; visualization. **Stuart Black:** Funding acquisition; methodology; supervision; writing – review and editing; conceptualization. **Andrew C. Kitchener:** Funding acquisition; conceptualization; resources; writing – review and editing. **Georg Hantke:** Resources; data curation. **Luke A. Stevens:** Methodology; investigation; formal analysis; writing – review and editing. **Lea J. Grayston‐Smith:** Methodology; investigation; formal analysis; writing – review and editing. **Phillip J. Baker:** Resources; writing – review and editing. **M. Alejandra Perotti:** Funding acquisition; conceptualization; writing – review and editing.

## CONFLICT OF INTEREST STATEMENT

The authors declare no competing interests.

## Supporting information


**Table S1.** Host and ectoparasite sample information. Metadata for host specimens and associated ectoparasites, including collection details, tissue types sampled and ectoparasite counts by host and feeding status.
**Table S2.** Isotopic values (mean ± SD) of fed and unfed ectoparasites. *δ* ^15^N and *δ* ^13^C values for fleas, lice and ticks, with mean differences (Δ) between fed and unfed individuals.
**Table S3.** Stable isotope values (*δ* ^15^N and *δ* ^13^C) of host tissues. Isotopic values (mean ± SD) of fur, whisker, muscle and spines from fat dormouse, red squirrel and hedgehog specimens, including tissue‐to‐tissue offsets (Δ).
**Table S4.** Parasite and host tissue isotopic values and offsets. *δ* ^15^N and *δ* ^13^C values (mean ± SD) for whole ectoparasites and host tissues, including calculated tissue‐specific and average offsets between fleas, lice, ticks and their hosts.

## Data Availability

The data that support the findings of this study are openly available in Dryad at https://doi.org/10.5061/dryad.qfttdz0t8 (Mortier et al., 2025).
